# Cognitive-Neural Effects of Brush Writing of Chinese Characters: Cortical Excitation of Theta Rhythm

**DOI:** 10.1155/2013/975190

**Published:** 2013-02-26

**Authors:** Min Xu, Henry S. R. Kao, Manlin Zhang, Stewart P. W. Lam, Wei Wang

**Affiliations:** ^1^Department of Linguistics, The University of Hong Kong, Pokfulam Road, Hong Kong; ^2^Department of Psychology, Sun Yat-sen University, Guangzhou 510275, China; ^3^Department of Psychology, Fu Jen Catholic University, New Taipei City 24205, Taiwan; ^4^International Society of Calligraphy Therapy, Hong Kong; ^5^Department of Psychology, The Fourth Military Medical University, Xi'an 710032, China

## Abstract

Chinese calligraphy has been scientifically investigated within the contexts and principles of psychology, cognitive science, and the cognitive neuroscience. On the basis of vast amount of research in the last 30 years, we have developed a cybernetic theory of handwriting and calligraphy to account for the intricate interactions of several psychological dimensions involved in the dynamic act of graphic production. Central to this system of writing are the role of sensory, bio-, cognitive, and neurofeedback mechanisms for the initiation, guidance, and regulation of the writing motions vis-a-vis visual-geometric variations of Chinese characters. This experiment provided the first evidence of cortical excitation in EEG theta wave as a neural hub that integrates information coming from changes in the practitioner's body, emotions, and cognition. In addition, it has also confirmed neurofeedback as an essential component of the cybernetic theory of handwriting and calligraphy.

## 1. Introduction

Shufa, or the brush writing of Chinese calligraphy, often regarded as a unique form of art in Chinese culture, is the writing of Chinese characters by hand using a soft-tipped brush. The heritage of Chinese calligraphy is traditionally used as a way to enhance an individual's self-reflection and cultivation. The study of calligraphy in the past has focused mainly on how to execute and appreciate it artistically by following the experiences of the great masters. During the past three decades, we have investigated the Chinese calligraphic behaviour from a scientific perspective and methodology. The theory-based research has established the effectiveness of practicing Chinese calligraphy in causing one's cognitive activation, perceptual sharpening, physiological slowdown, and emotional stability. The advent of cognitive neuroscience has inspired and facilitated the contemporary research into some cortical correlates of the execution of handwriting and calligraphy. Encouraging results have been reported in recent years.

## 2. Cybernetic Theory of Handwriting and Calligraphy

### 2.1. Sensory Feedback: The Behavioral Basis of Handwriting

A sensory feedback theory of handwriting was proposed and tested a long time ago [1, 2]. It incorporated various concepts of neurophysiology in the guidance and regulation of human movements and sensory feedback. The act of writing is based on immediate sensory feedback from the different component movements of the hand and arms and from the marking action of the pen point. The writer obtains reactive feedback from the movements of the hand itself, visual feedback from the action of the writing instrument, and a persisting graphic or operational feedback from the records of writing on the paper. These sources of motion-regulated stimulation are made coherent by the fact that feedback control is focused on the action of the writing point in producing graphic records. All levels of reactive, instrumental, and operational control are expressed in this basic marking action of the focal writing point. The graphic record of the writing creates a persisting visual feedback of the immediate writing performance and thus provides a basis for the possible feed forward regulation and prediction of writing to be formed in the immediate future during the writing process. This feedforward guidance determines the temporal continuity in tracking movements and the behavioral efficiency in handwriting and penmanship. Previous studies of the importance of visual feedback and the maximal writing efficiency through a combined instrumental and operational feedback have been reported [3, 4].

### 2.2. Bioemotional Feedback: The Physiological Basis of Handwriting

In addition to the sensory feedback conceptualization of handwriting of the 60s, we further investigated some physiological correlates of handwriting by looking into the real-time bodily changes that took place when one engaged in this graphological act, this time by way of writing Chinese characters with a soft-tip brush, and obtained fruitful results and insights into this behavior. The general conclusions of this series of experiments confirmed an overall physiological slowdown in the practitioner's heart rate, respiration, blood pressure, skin conductance, and so forth in the course of the writing act [5]. The direct outcome of such physiological changes as well as the overall physical quiescence evoked by the 3D brush writing was the sensory feedback state of emotional relaxation, calmness, tranquility, and peace of the mind on the part of the practitioner, which offered a psychological incentive or a feedback-driven source for feed-forward control and execution of the characters. In other words, one reason for the practitioner to be motivated to continue the writing was that during this act he enjoyed the feedback of physiological slowdown and the soothing and relaxing states of emotions and affect, so that he was naturally attracted and reinforced by his ongoing physical changes and sensory experiences during calligraphic execution. We considered this behavioural phenomenon a new form of feedback that is additional to the indices of behavioural feedback concept we proposed in the 60s. This outcome added a new dimension of bioemotional feedback to our theory of handwriting and calligraphy [6].

### 2.3. Cognitive Feedback: The Interface between Characters and Thought Activities

Arising from researching the effects on the practitioners as a function of the visual-spatial variations of the characters, it came to our realization that these cognitive contents in terms of character geometricity were so powerful, upon repeated replications, that this factor became our last dimension in our theory for handwriting and calligraphy. This new dimension, called cognitive feedback, refers to the subjective experiences of heightened attention, alertness, and quickened response capacity that occurred during the writing action as a direct result of the character-cognition interactions. This form of feedback draws its impact from the visual-spatial variations of the character executed in the writing process [7–9]. Long-term effects of this interface are often evoked and demonstrated in enhanced visual space ability, abstract reasoning, spatial reasoning, and short-term memory. Something having to do with the cortical stimulation and arousal may be related to these psychological phenomena. Thus, up to this point, the cybernetic theory of handwriting and calligraphy now comprises behavioural feedback, biofeedback, cognitive feedback and neurofeedback vis-a-vis character structural specificity and motor control as the core components of this unique form of graphic behavior. The concept of neurofeedback here refers to a prediction that the information from the three other sources would be centralized and reflected in some cortical stimulation during writing. This was one of the goals of the present experiment.

### 2.4. Character Geometricity in Chinese Calligraphy

Moving into the 1990s, we explored in detail the interaction between character and the calligraphic act of writing in order to study the interface between the shapes and forms of Chinese characters and its consequential effects on the writer's psychophysiological functions during writing. Soon we began to realize that the visual-spatial variations among the characters did have a powerful impact on changes and alteration of writer's bodily states. We further confirmed the structural basis of character geometricity and its corresponding effects also on such physical changes. A vast amount of data convinced us that the core of changes in the writer's behavioural as well as bioemotional changes were directly attributable to the visual-spatial variations of the characters shapes and forms. Therefore, at this stage of theory development, we suggested yet another dimension to the cybernetic theory of handwriting and calligraphy, that is, the visual-spatial properties of the character construction as a key component in handwriting that is related to and caused the observed variations of the writer's behavioural, emotional, cognitive and neurocognitive responses [7, 10, 11]. This new dimension of character geometricity has enriched, broadened, and consolidated our general cybernetic theory of handwriting and calligraphy. The following section describes in brief some major principles concerning this character-writing interface.

### 2.5. Cybernetic Principles of Character Writing

The writing of Chinese characters can be conceived of as a process of visual-spatial structuring of the elements of characters. They are written within an imaginary, subdivided square in which the execution of strokes into character (Jiezi), shaping of the character (Jieti), and the spacing and framing of the character (Jianjia) occurs. Jiezi refers to the basic formation of strokes within a given character and their structural interrelationships. Jieti is a process of organizing the various strokes to conform to the style of the character, and Jianjia is the layout and spacing of characters, as well as their positions in columns and rows [12]. The purpose of a shaping character is to ensure its coherence and autonomy relative to other characters in a given writing context. The formation of a character involves inscribing it uniformly in a square and then centering it, irrespective of its form or relative size.

Chinese writing, especially with a brush, can be conceptualized as an act involving the whole body of the writer in which cognitive planning, organizing and processing of the visual-spatial patterns of the character take place. Motor control and maneuvering of the brush following the character configurations involve the whole body projected relative to the geometry of each character. The activity of brush writing is essentially an external projection and execution of the writer's internal cognitive images of the character. There is therefore an integration of mind, body, and character inter-woven in the dynamic calligraphic process. This intimate relationship underlies the interactive effects of character writing on the mind and the body of the writer, as does the spatial organization of the character in writing. 

Central to the perceptual organization of the character from within the calligrapher's cognitive experience are some properties underlying the visual-spatial structure of Chinese characters. The visual or imaginary frame can be analyzed from the perceptual elements of balance, space, shape, form and movement. On a more visual-spatial level, several topological principles of visual perception are pertinent to the cognitive map of the character produced in and by the act of writing. These include the presentation of global views and detailed parts of objects, connectedness, inside-outside relationships, number of holes, colinearity, size, orientation, and symmetry. In the process of brush writing or calligraphic gesturing, the writer's perceptual shaping of the character is affected by the patterns within the character and cause his perceptual, cognitive, and bodily conditions to engage in corresponding adjustments and representations. This dynamic process, incorporating character writing within the practitioner's body and mind, would result in the course of calligraphic production in his perceptual, cognitive and psychophysiological responses varying in respect of the visual-spatial configurations of the strokes in the character being written. Characters sharing these visual-spatial properties are predicted to have a greater impact on the practitioner than those characters sharing less of these properties.

A series of experiments has been conducted to test the hypothesized relationship between the effects of brush writing and the perceptual configurations of the characters [13]. Observations on effects of global, detailed and figure and ground variations of visual-spatial configurations as well as other topological properties provided positive support for a theoretical formulation of the perceptual-cognitive-physiological model of Chinese brush character writing and also confirmed the significance of considering character characteristics from topological principles of visual perception.

A second set of experiments involved specific selection of characters conforming closely to specific visual-spatial properties of stroke and character structure and investigated how the reproduction of these characters using a brush might affect the behavioral responses of the subjects. The spatial properties of the characters investigated varied in terms of global detail (i.e., the presentation of the whole or parts), holes-no-holes, colinearity, symmetry, and directionality in orientation. Results strongly support the stated hypotheses and lend significance to the basic theoretical formulation of character writing [7, 11, 14].

The overall findings in these studies provided encouraging confirmation of the feasibility of a mind-body-character interface or psychogeometric model of character writing. A conceptual framework has been advanced to highlight the above observations with a systematic analysis of the character's structural components and their role in the act of Chinese character writing [10]. 

Calligraphy involves the visual-spatial relations and the numerous and diverse ingredients of Chinese character structure and directly connects and promotes connections of cranial nerves. Stimulation of the cerebrum should be able to strengthen with the multidimensional visual-spatial relations, combination, variation, shape differences, and so forth. Early research discovered that Chinese character writing has the function of brain cognitive activation, which may be concerned with the functional plasticity of human cerebral cortex. When processing the visual-spatial configurations of Chinese character forms at writer's cortical level, the writing activity constitutes the reproduction, restoration, and the processing of visual perception and therefore can initiate the activation function to be advantageous to the operation of cognitive activity and further mold and enhances the functions of related cortical substrates [10]. 

### 2.6. The Search for Neurofeedback in Handwriting

In the course of developing the cybernetic theory of handwriting and calligraphy, we have introduced the three types of behavioral feedback, the sensory feedback, bioemotional feedback, and cognitive feedback as simultaneous responses evoked in the writing process. But a theory of this particular nature will not be complete without also demonstrating the neural mechanisms that underline and integrate all three sources of feedback in the central nervous system. Early discussions of this issue were first introduced by Smith and Murphy (1963) [2], who foresaw the possible link of a neuronal network in the brain directly interfacing and connecting with the changes and variations of the writing materials. Unfortunately, little research has followed this conceptual contribution until recently when we focused on such a neural correlate with the ERP methodology [11] and behavioral measures [15]. Despite these preliminary experiments in this direction, we were keen to pursue deeper into this line inquiry with the aid of contemporary neuroscience theories and methodologies in order to test and validate our theoretical predictions concerning the functional role of handwriting and calligraphic practice in stimulating, activating and, evoking changes in cortical substrates and functions.

 In this study we were interested in the neurocognitive correlates of calligraphy writing for a more fundamental understanding of the character-brain-cognition interactions as well as its likely effects on functional neurophysiologic changes in the brain. By designing this research, we also wanted to find yet a further component to the cybernetic theory of handwriting, that is, a central mechanism of neurofeedback that would integrate simultaneously the sensory feedback, bioemotional feedback and cognitive feedback all into a central coherent state that is reached through the practice of handwriting or calligraphy.

As evidence accumulated that calligraphy treatment is therapeutically effective, one question to be answered is the reasons behind all these positive effects of the practice of Chinese calligraphy. Writing involves perceptual processes like the visual recognition of the stimulus and highly controlled and well-coordinated sensorimotor movements, which is further complicated by the softness of the brush. Such a sophisticated series of actions requires intensive attention and concentration from the writer. He or she has to reduce both external disturbances (e.g., noise) and internal disturbances (e.g., emotional fluctuations and breathing) to a minimum. This high level of attention is evidenced when the heart rate decelerates, which represents a “somatic quieting.” This deceleration and the intensive involvement of cognitive resources result in an improvement of memory capacities in recall and recognition as well as mental capacities for other functions [16–18]. Concurrent to these states, some cognitive effects occur in relation to a person's attention and concentration as a function of the geometric characteristics of the writing script, which contribute to the facilitative benefits observed in one's cognitive activities [19]. Findings of an event-related potential (ERP) study offered further evidence for the activation of human brain during CCH, in which the postwriting cortical arousal and activation are shown to be significantly greater than prewriting baseline for the calligraphy writing subjects, whereas no such changes were observed for the controls [11].

However, the extent to which brain activity can be changed through calligraphy training has yet to be adequately addressed. Studying the neural basis of calligraphic writing enables us to detect even subtle changes in the brain system that are associated with the writer's mental state and provides us with fundamental evidence for the application of this training program. For the present study, the use of EEG to assess the effects of calligraphy training on brain function was a logical choice for the research question. Of particular interest are changes in theta wave of frontal midline after the calligraphy treatment. The use of EEG measures was prompted by their promotion as a more direct measure of attentional states, their success in differentiating varying states of attentional focus, and their stability and reliability over time [20]. Ample evidence suggests that the EEG is a valid measure of the functional state of the brain [21]. 

### 2.7. A Neuro-Cognitive Marker: The Theta Rhythm in Frontal Midline

Theta rhythm is the “rhythm with a frequency of 4 to 8 Hz.” It has at least two manifestations in human brain [22]. The first is characterized by a widespread scalp distribution and being observed during drowsiness and states of low-level alterness. The second type is often referred to as frontal midline theta (Fm theta), which was first observed and named by Ishihara and Yoshii in Japan in the 1960s. Fm theta was considered to include theta rhythms that are maximal in the frontal midline region (F3, Fz, F4) [23]. 

Fm theta activity has been observed in many tasks including mental calculation [24, 25], working memory and learning [26–28], and meditation [29–31]. The majority of studies investigating Fm theta during working memory tasks have shown an increase in theta activity with increasing working memory load and task difficulty [27, 32–35]. Aftanas and Golocheikine [29] noted an increase in theta (4–6 Hz) and alpha 1 (6–8 Hz) power over frontal and midline regions in long-term meditators compared to controls and short-term meditators. In addition, it was reported that Fm theta was reduced in normal aging subjects [36]. Furthermore, the appearance of Fm theta reflects a feeling of relief from anxiety. Individuals exhibiting greater Fm theta activity tend to have lower state and trait anxiety scores [37]. The Fm theta correlates with changes in anxiety levels induced by antianxiety drugs, which suggests that the appearance of Fm theta is influenced by the drugs and that the relief from anxiety might be involved in the appearance of Fm theta [38–40]. Some researchers explained that increased Fm theta in meditation is associated with reported decreases in anxiety levels and other positive emotional experiences [29].

In sum, Fm theta is recognized as distinct theta activity in the frontal midline area and reflects mental concentration as well as state of relief from anxiety [31]. This “relaxed concentration” state, as a previous research shows, is the very state during calligraphy handwriting. Therefore, we hypothesized that subjects who were administered calligraphy training would demonstrate significant increase in theta rhythm of frontal midline compared to participants in a control condition. 

In our previous research, global style of character presentation led to better psychological indexing than the detailed style of character presentation, while linear style of character presentation proved to be less favorable than the nonlinear style of stroke presentation, as shown by a reduction in heart rate. These observations provided positive support for a theoretical formulation of the perceptual-cognitive- physiological model of Chinese brush character writing and also confirmed the significance of considering character characteristics from the topological principles of visual perception. These observations may be also indicated by the patterns of EEG evoked during writing characters with different visual-spatial properties. 

Therefore, on the basis of the above background, the aims of the present study were (1) to examine whether Fm theta wave changes through a 10-day calligraphy training and how, (2) to further investigate the role of visual-spatial properties of the characters in EEG wave patterns during brush handwriting, and (3) to explore the role of theta wave as a neural marker of integration of the states of the calligraphy-evoked physical and cognitive responses of the writer during writing. We predict that calligraphic training would lead to an increase in the Fm theta waves and that writing characters with global and nonlinear style can evoke greater among Fm-EEG waves than those with detailed and linear style. 

## 3. Materials and Methods

### 3.1. Participants

A total of 16 undergraduate students participated in this experiment on a voluntary basis. They were assigned to the calligraphy group or the control group, 8 participants in each group. One of the participants had experience in Chinese calligraphy for more than 3 years, while the remaining participants had little experience. Informed consent was obtained from all of them in written form after the experimental procedures had been fully explained.

### 3.2. Materials

The calligraphy writing materials were those characters with visual patterns designed on the basis of a visual-spatial geometric system. They were of varying levels of properties such as closure, symmetry, linearity, parallelism, connectivity, and orientation. Forty characters were chosen for ten days training, with each characters appearing twice once in detailed style and once in global style. Of particular interest are EEG waves evoked by characters with detailed versus global style and linear versus nonlinear style. The global style character is constructed with an outer contour for its interconnected as well as independent strokes, so that the character exhibits a visual closure of the space. The detailed style character lacks such features but has only solid strokes throughout. The linear style character is made up with only straight strokes, whereas the nonlinear style character has only curves or wavy strokes. 

### 3.3. Apparatus and EEG Recording

The ProComp2 system (Thought Technology Ltd.) equipped with one EEG channel was adopted for data acquisition. This battery-operated portable unit worked with a laptop PC. EEG recordings were taken under four conditions: eyes open, a color test, eyes closed, and writing/sitting quietly. A gold-plated electrode designed like a snap-on button was positioned in frontal midline (Fz) according to the international 10–20 system and earlobes were used as a reference site. Sample rate for the EEG channel was 256 per second. The BioGraph Infiniti program also includes a global notch filter function, which reduced the effect of electromagnetic interference on the signals.

### 3.4. Procedure

The calligraphy group undertook a standard prewriting test of 6 min and 43 sec once before and once after the treatment schedule. Calligraphy therapy protocol was used for the calligraphy group. This daily treatment was 20 min per session for 10 sessions consecutively for each subject. The controls did not engage in any actual treatment but quiet sitting for 20 min between pre- and posttest and they had to come to take the measure on the first and the tenth day. The treatments were conducted in a research laboratory.

### 3.5. Data Analysis

The mean peak to peak amplitude of theta (4–8 Hz) was used for analyzed. To ensure that the data for the writing stage were indeed those of writing act, we used the previous 10 seconds data of writing each character, considering that the writers, for the most part, would not finish writing a character within ten seconds. We first compared theta wave between day 1 and day 10 for the 2 groups. Data were submitted to a 2 × 2 mixed design ANOVA, in which group (two levels: calligraphy group and control group) served as the between-subjects variable, while time (two levels: day 1 and day 10) served as the within-subjects variables. Then we further compared theta wave of three stages: before writing, during writing, and after writing within a single day. 

**Figure 1:**
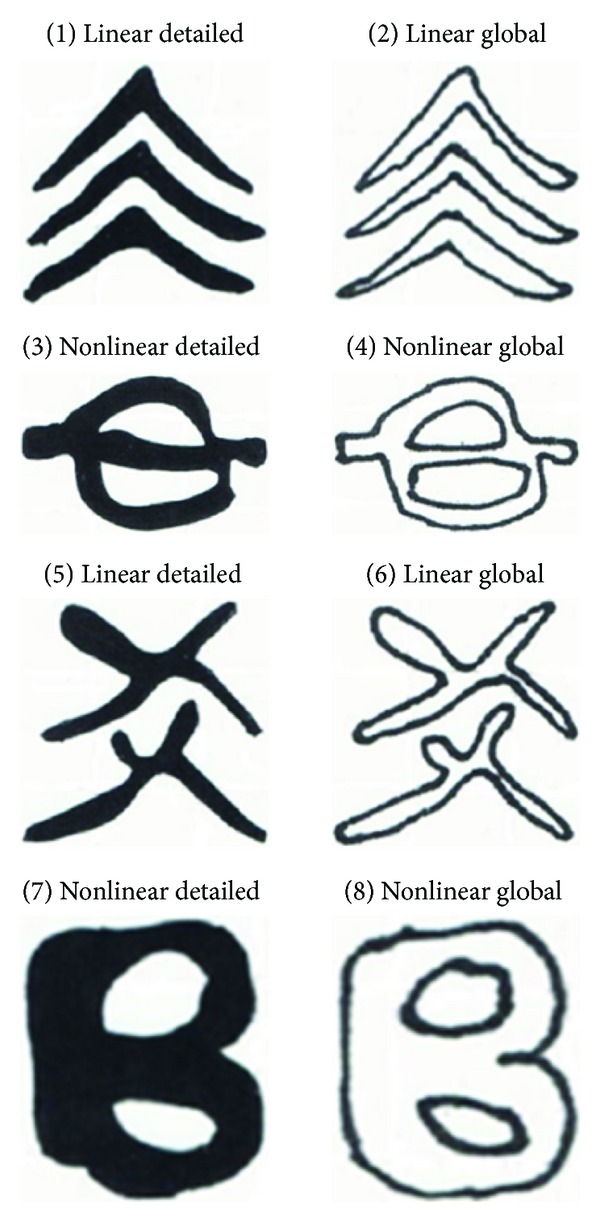
Character varying in visual-spatial properties.

To investigate the role of visual-spatial properties of the characters in causing EEG wave patterns, we selected eight characters (as shown in Figure 1), whose linear/nonlinear features were most prominent among the writing materials. Pairwise *t*-test was performed to compare EEG waves evoked during writing by the characters with detailed style to the corresponding ones with global style and also the linear characters with nonlinear ones. In this analysis, we include alpha wave besides theta wave because evidence has suggested that both frontal midline theta and alpha reflect a state of concentration and positive emotion experience [29]. The analyses were two tailed, with the significance level set at 0.05. Data analysis was carried out using SPSS 16.0.

## 4. Results

**Figure 2:**
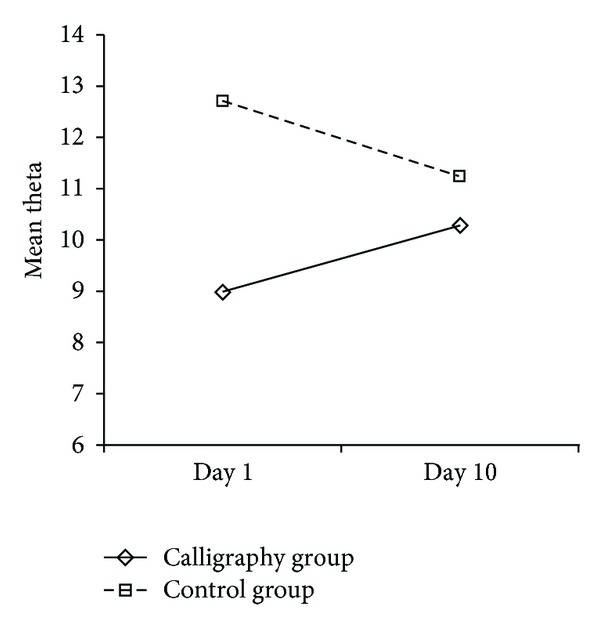
Theta mean changes between day 1 and day 10 for the two groups during prewriting.

For repeated measures ANOVA, Pillai's trace is reported. During prewriting, though the main effect of time did not reach significance, there's a significant time × group interaction in prewriting theta wave, *F*(1, 14) = 4.991, *P* < 0.05 (Table 1 and Figure 2), which indicated difference between the two groups in theta changes between day 1 and day 10.

**Figure 3:**
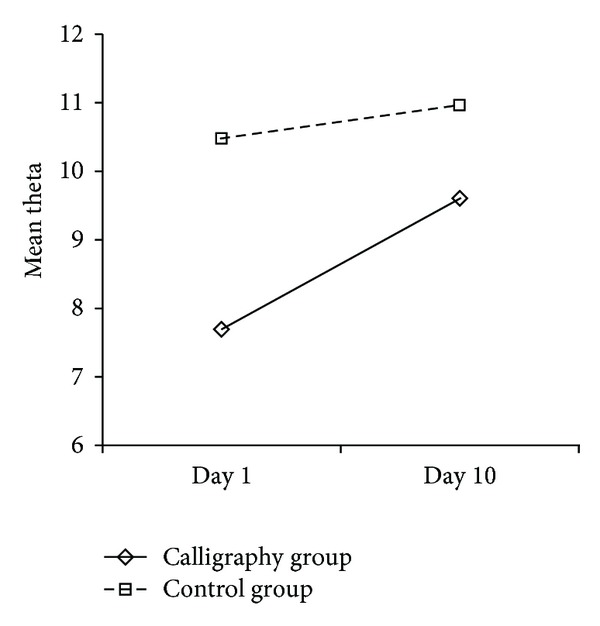
Theta mean changes between day 1 and day 10 for the two groups during writing/sitting quietly.

The main effect of time in during-writing theta wave attained significance (*F*(1,14) = 12.211, *P* < 0.01), and it was qualified by a marginally significant time × group interaction (*F*(1, 14) = 4.414, *P* = 0.054). That means there's a trend of difference among the two groups in theta changes between day 1 and day 10 (Table 1 and Figure 3). When theta data were treated respectively for each group, results showed that during writing there was a significant increase of theta in the calligraphy group (*F*(1, 7) = 12.829, *P* < 0.01), whereas theta mean of control group (sitting quietly) showed no significant change (*F*(1, 7) = 1.245, *P* > 0.05).

Though the two groups of participants showed different amounts of theta waves during prewriting and during writing/siting periods on day 1, distinct patterns of changes in the two groups demonstrated that calligraphy training is associated with increased amount of Fm theta waves.

**Figure 4:**
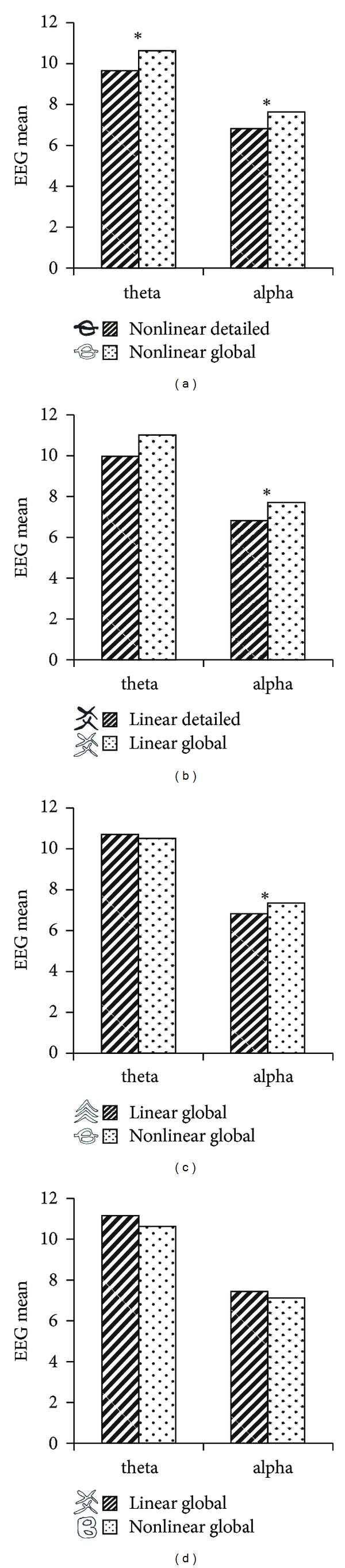
Comparison of theta and alpha waves evoked by (a) nonlinear-detailed versus nonlinear-global characters, (b) linear-detailed versus linear-global characters, and (c) and (d) linear-global versus nonlinear-global characters. Note: *indicate significant difference at *P* < 0.05.

Finally, we performed pairwise *t*-test in the comparison of frontal midline theta and alpha waves evoked by characters with varying visual-spatial properties (Figure 1 and Table 2). Results showed that characters with global style evoked greater theta and alpha waves than detailed characters (Figures 4(a) and 4(b)). Comparison between linear and nonlinear characters showed significant difference of alpha wave in one pair of linear-global and nonlinear-global characters (Figures 4(c) and 4(d)). 

## 5. Discussion

### 5.1. Implications of Increased Frontal Midline Theta Evoked by Calligraphy Training

In this study, we examined the effects of calligraphy training on theta wave generated in frontal midline. The mean value of theta in the calligraphy treatment group, as predicted, showed a considerable increase after a ten-day calligraphy training. This change reflected the relaxed and concentrated state evoked by calligraphy.

The act of brush handwriting results in physiological slowdown and relaxation, as indicated by the changes in heart rate, blood pressure, respiration, and skin temperature after calligraphic writing. Taken together with the findings of calligraphic effects on brain activity and cognitive functions, these outcomes suggest that calligraphic writer's body is under a relaxed state while the mind is under an apprehensive and intensive state. These two seemingly contradictive physiological phenomena work together in a calligraphic act. The present study disclosed the role of frontal midline theta as a neural-cortical hub that integrates these calligraphy-evoked physical and cognitive responses observed. A concentrated and relaxed state has also been found during such activities as mediation, memory tasks, and calculation, but none of these activities involve any motor tasks like calligraphy handwriting. The theta evoked during writing explains the unique state of mind-body coherence that was reached in our early research.

Theta wave is important for integrating different brain regions into networks [41, 42], and thus increased frontal theta may also indicate improved network function. Meanwhile, frontal midline theta may constitute a neurophysiological marker of changes in cognitive networks [36]. If this is the case, increase of theta wave in the present study offers fundamental neurophysiological evidence for the amelioration of cognitive ability, especially in children with mental retardation as well as patients with Alzheimer's disease. In addition, considering the well-established link between frontal midline theta and positive emotional experience [29], increased frontal midline theta observed in this study offers deep insight into the neurological mechanism underlying the positive effects of calligraphy training on affective variables of anxiety and depression, as shown in previous studies. This observation has provided clear support to the role of frontal midline theta as a neurological marker that integrates the evoked states of the body and the mind through, respectively, biofeedback and cognitive feedback during writing. This neurofeedback mechanism takes place rather unperceptively on the part of the writer but is through the hubbing effect of the calligraphy-evoked physical and cognitive responses observed. This finding has enriched the cybernetic theory of handwriting and calligraphy by further adding “neurofeedback” as the cortical center of all other feedback sources that are processed during the writing act. At this stage, the cybernetic theory of handwriting and calligraphy is confirmed as a multifeedback behavioral system which consists and integrates the sensory feedback, the bioemotional feedback, the cognitive feedback, and the neurofeedback in a dynamic process of graphic production.

### 5.2. Influences of Characters' Visual-Spatial Variations on Frontal Midline EEG

The variety of EEG waves takes place naturally and simultaneously in the course of writing; it shows a total involvement of the mind and the body in its production. The character-EEG correlations, especially the consistent differences of EEG amount evoked by global and detailed characters, confirm the role of the visual-spatial properties of the character in causing varying EEG wave patterns. These findings have implications toward designing different character forms for promoting general cognitive health as well as for the treatment of cognitive disabilities or disorders.

A most important factor leading to calligraphic effects on the brain activity changes in this study, and also the facilitative benefits in one's cognitive functions in previous studies should be the contents of writing that involves the topological properties as well as the gestalt properties. Abundant psychogeometric features of script formation including studies have confirmed the effects of visual-spatial properties of Chinese characters on perceptual, cognitive and psychophysiological responses of practitioners [9, 11, 19]. The writing materials in the present study were those characters with varying levels of such properties as closure, symmetry, linearity, parallelism, connectivity, and orientation. In the process of brush writing or calligraphic gesturing, the writer's perceptual shaping of the character is believed to be affected by the patterns within the character and cause his perceptual, cognitive, and bodily conditions to engage in corresponding adjustments and representations. Here, the geometric characteristics of the writing script have also come into play to change the cortical activities. Additionally, findings from researching Chinese handwriting can be generalized to alphabetic and other scripts, since all scripts share certain geometric properties in their construction and the basic principles of drawing and handwriting are common between Chinese and English [13, 43, 44]. These results have validated the conceptualization of character geometrics as a fundamental influence in the perceptual, cognitive and motor processes of handwriting that cause varied forms of psychological effects of this practice. The stated relationship between character and writing interface has been, in this research, established with the demonstration of the first cognitive-neural evidence in EEG theta in the frontal midline of the cortex.

### 5.3. Intake-Rejection Hypothesis to Explain the State of Calligraphy Handwriting

The theoretical exploration about the relationship between calligraphy handwriting and heart rhythm can be set about to Lacey's (1967) [45] intake-rejection hypothesis, which states that the direction of change in heart rate was dependent on the nature of the task to which subject was exposed: intake of information (external information processing), for example, empathic listening and visual attention, results in decrease of heart rate and increase of the skin conductance response, whereas rejection of information (internal information processing), for example, reverse spelling and mental arithmetic, results in increase of both HR and SCR. Cardiac deceleration was an indication of the intention to note and detect external events [46]. The fact that increases in heart rate and blood pressure, under appropriate conditions, produce inhibition of a wide variety of central neural processes led them to hypothesize that cardiovascular activity may have a special role both as a correlate and a determinant of behavior [47]. Lacey further elaborated on the neurophysiological evidence for this hypothesis mainly based on the acute physiological preparations in infrahuman mammals [45]. They concluded that decreases in heart rate and blood pressure would be disinhibiting and would produce “a net increase in [cortical] excitation: a lowering of threshold, a prolongation of the impact of stimuli, an increase in spontaneous activity, and the like.” Increases in heart rate and blood pressure, on the other hand, would result in raised thresholds for sensing stimuli, a less enduring impact of a stimulus, a decrease in spontaneous activity, and so on. Chinese character writing is actually a kind of external projection and execution of internal cognition image of Chinese character. The visual-spatial properties of the character affect the cognitive and motor activities of the writer during writing [14]. At this point, it is reasonable to take calligraphic writing as a process of intaking information offered by Chinese characters, resulting in a decrease of HR, increase of skin conductance response, and further increase in cortical activity.

Chinese calligraphic handwriting involves a 3D motion because of the softness of the brush tip. The key point to handle a Chinese writing brush is to keep it flexible easy to be turned, and it is important to control the direction of the tip as well as the speed of moving the brush [48]. Owing to the differences in the character sizes, the thickness of the strokes, the features, and direction of a particular style of writing a smooth and effective adjustment of the movements of the fingers, the wrist, the arm, and the shoulder is essential [17]. Therefore, soft-tipped calligraphy, compared to writing with hard-tipped tools (pens, pencils, etc.), demands greater attention to the motor control and the manoeuvring of the brush. The writer allocates the required attention to the 3D task and thus better expels the incoming irrelevant stimulus and information, according to the attentional resource allocation theory which proposes that the attentional resource for processing information is finite and that allocating additional attention to a task would reduce the resources available for other concurrent tasks. Therefore, the characteristics and requirements of brush handwriting cause heightened attention and concentration on the practitioners.

### 5.4. Cultural Implications of the Present Study

The findings of this present research have provided empirical evidence to the foundations of a cybernetic theory of handwriting and calligraphy as well as contributed to the beginning of future research into the neuro-cognitive mechanisms underlying the dynamic action of character writing. As is evident, the most important areas of calligraphic contribution rest in those disorders or deficiencies that are associated with some aspects of man's cognitive functions, moods, emotions, and motor behaviors. On the basis of this and past studies, we are optimistic that this new therapeutic system will flourish and be developed for broader applications in the future and to users of other writing systems as well. 

Handwriting is a complex act of perceptual, cognitive and motor activity in which a direct interface between language and cognition, script and writing, as well as character and cortical responses through the soft-tipped brush writing for calligraphic performance. This study has taken the first look at the neuro-cognitive effects and the neural mechanism of brush character writing. The results are confirmative of the views and expectations of the cybernetic model of handwriting and calligraphy [1] as well as the set of psycho-geometric principles that predict the perceptual, cognitive, and physiological effects of brush writing Chinese characters [10]. The varied EEG wave patterns, found to vary with the visual-spatial properties inherent within the characters, have provided the first cortical evidence of this relationship stated from our theoretical predictions.

These preliminary findings are also significant from several broad cultural perspectives. Firstly, handwriting is not only the simplest form of man-machine or man-tool system but also an act embracing man's perceptual-cognitive-motor activity that has been shown to cause differentiating physiological, cognitive, and cortical responses on a real-time, simultaneous, and feedback-driven basis. We have found this practice to be able to slow down and relax our bodily functions while at the same time heighten and activate our cognitive and neural activities. Increased EEG theta has been confirmed to be the neural core that integrates these two contradictory behavioral phenomena of the body and the mind as well as a hub for neural networking of these responses with other brain functions. This suggests a biological basis for calligraphy therapy to be an effective form of behavioral treatment. Since the research was designed to tackle a general system of handwriting of which calligraphy is a unique Chinese form, the observations gathered would have implications and generality for the behavior of handwriting in other writing systems.

Secondly, one primary interest of the present study was the prediction of a direct relationship between geometric forms and neural EEG changes through a spectral analysis. The results have disclosed a direct impact of visuospatial properties of Chinese characters on corresponding and distinguishing EEG wave variations. This finding offers a new perspective toward examining the basis of character-brain interface through brush writing in other linguistic forms or writing systems. It presents also an opportunity of studying this relationship between functional neuroplasticity and the dynamic act of brush writing of varying character forms. We previously suggested this direction of handwriting research some 10 years ago and are now feeling gratified that the current neuroscience contributions are helping and leading the way for us to move toward that direction [10].

Thirdly, recent studies [49, 50] have suggested that, on the basis of studying dyslexia, Chinese language learning is facilitated by writing and that for alphabetical language is, in contrary, by phonological processing. The findings of the present study offer direct neurocognitive evidence to the authors' proposition. Moreover, the research outcomes have, further, broadened its generality from writing Chinese characters to that of other writing systems in other cultures. Therefore, this study may be able to offer further insights into the possible role of visual-spatial properties of the characters in enhancing language learning, cross-linguistic education, the design of educational materials and linguistic therapy and rehabilitation.

Fourthly, we believe that the present outcomes have made a modest contribution to our understanding of the action dynamics of human language and thought processes. Unlike reading, which differs in script and sound components among the various scripts, handwriting in all writing systems shares the same perceptual, cognitive, and motor processes because at the basic level of analysis, scripts only vary in character shapes, forms, and structure across languages and cultures. The psycho-geometric principles of character construction in general would provide a unifying tool of comparison across the scripts. Our past and recent experience of clinical and treatment cases, in real-life implementation of these psycho-geometric principles as the core of the cybernetic theory of handwriting and calligraphy, have amply established the clinical validity of our predictions. The conclusions of this research suggest that its significance is not only pertinent to Chinese brush handwriting of calligraphy as a unicultural activity, but has also cross-cultural generality in theory and practice. It should be possible to look forward to future developments for methods and technologies specifically designed for enhancing graphonomic learning, practice, and remediation as well as for facilitating cognitive health and therapeutic interventions. This research has made a modest contribution in this endeavor. 

## 6. Limitations

One limitation of the present study is that we used one channel EEG, which was placed to Fz according to the International 10–20 system. Fm theta was found to be maximal also in other frontal midline regions including F3 and F4 [23]. Using multichannels, therefore, may provide more robust and accurate estimate of EEG rhythms. Another limitation is that we included eight characters in examining the influences of characters' visual-spatial properties on EEG due to the limited number of characters with designated variations of visuospatial features and without confusion of other properties. Future work is needed to extend our finding based on more character samples. 

## 7. Conclusions

The overall results of this study in the context of a cybernetic theory of handwriting and calligraphy has given additional credence to the biological basis of calligraphy therapy as an evidence-based system of behavioral treatment and rehabilitation. The many clinical studies and control trials that we have conducted can now have greater pool of theoretical, empirical, and clinical trials foundations to testify the successes of our professional applications over the years of this system of behavioral therapy, particularly in the areas of cognitive maintenance, cognitive treatment, and cognitive rehabilitation and, generally as well, in such other areas of health promotion and disease intervention as psychosomatic disorders, emotions, psychiatric conditions, and behavioural problems.

## Figures and Tables

**Table 1:** Mean theta wave in calligraphy group and control group during prewriting and writing periods.

	Calligraphy group		Control group	
	mean (SD)		mean (SD)	
	Day 1	Day 10	Day 1	Day 10
Prewriting	8.98 (3.59)	10.27 (3.09)	12.71 (2.75)	11.24 (2.25)
During writing	7.69 (1.83)	9.60 (2.16)	10.48 (1.24)	10.96 (2.16)

(SD: standard deviation).

**Table 2:** Mean theta/alpha wave during writing the four pairs of characters.

	Mean theta (SD)	Mean alpha (SD)		Mean theta (SD)	Mean alpha (SD)
Nonlinear detailed	9.61 (2.73)	6.79 (2.50)	Linear detailed	9.99 (3.52)	6.9 (3.12)
Nonlinear global	10.58 (3.17)	7.61 (2.86)	Linear global	11.03 (4.35)	7.72 (3.39)

Linear global	10.64 (3.55)	6.78 (2.78)	Linear global	11.02 (4.41)	7.37 (3.37)
Nonlinear global	10.48 (3.28)	7.29 (2.76)	Nonlinear global	10.6 (2.89)	7.01 (2.99)

(SD: standard deviation).
